# Efficacy of mass drug administration with ivermectin for control of scabies and impetigo, with coadministration of azithromycin: a single-arm community intervention trial

**DOI:** 10.1016/S1473-3099(18)30790-4

**Published:** 2019-05

**Authors:** Lucia Romani, Michael Marks, Oliver Sokana, Titus Nasi, Bakaai Kamoriki, Billie Cordell, Handan Wand, Margot J Whitfeld, Daniel Engelman, Anthony W Solomon, John M Kaldor, Andrew C Steer

**Affiliations:** a The Kirby Institute, UNSW, Sydney, NSW, Australia; b St Vincent's Hospital, Sydney, NSW, Australia; c Murdoch Children's Research Institute, Melbourne, VIC, Australia; d Clinical Research Department, Faculty of Infectious and Tropical Diseases, London School of Hygiene & Tropical Medicine, London, UK; e Hospital for Tropical Diseases, London, UK; f Ministry of Health and Medical Services, Honiara, Solomon Islands; g Centre for International Child Health, University of Melbourne, Melbourne, VIC, Australia

## Abstract

**Background:**

In small community-based trials, mass drug administration of ivermectin has been shown to substantially decrease the prevalence of both scabies and secondary impetigo; however, their effect at large scale is untested. Additionally, combined mass administration of drugs for two or more neglected diseases has potential practical advantages, but efficacy of potential combinations should be confirmed.

**Methods:**

The azithromycin ivermectin mass drug administration (AIM) trial was a prospective, single-arm, before-and-after, community intervention study to assess the efficacy of mass drug administration of ivermectin for scabies and impetigo, with coadministration of azithromycin for trachoma. Mass drug administration was offered to the entire population of Choiseul Province, Solomon Islands, and of this population we randomly selected two sets of ten sentinel villages for monitoring, one at baseline and the other at 12 months. Participants were offered a single dose of 20 mg/kg azithromycin, using weight-based bands. Children weighing less than 12·5 kg received azithromycin oral suspension (20 mg/kg), and infants younger than 6 months received topical 1% tetracycline ointment. For ivermectin, participants were offered two doses of oral ivermectin 200 μg/kg 7–14 days apart using weight-based bands, or 5% permethrin cream 7–14 days apart if ivermectin was contraindicated. Our study had the primary outcomes of safety and feasibility of large-scale mass coadministration of oral ivermectin and azithromycin, which have been previously reported. We report here the prevalence of scabies and impetigo in residents of the ten baseline villages compared with those in the ten 12-month villages, as measured by examination of the skin, which was a secondary outcome of the trial. Further outcomes were comparison of the number of all-cause outpatient attendances at government clinics in Choiseul Province at various timepoints before and after mass drug administration. The trial was registered with the Australian and New Zealand Trials Registry (ACTRN12615001199505).

**Findings:**

During September, 2015, over 4 weeks, 26 188 people (99·3% of the estimated population of Choiseul [n=26 372] as determined at the 2009 census) were treated. At baseline, 1399 (84·2%) of 1662 people living in the first ten villages had their skin examined, of whom 261 (18·7%) had scabies and 347 (24·8%) had impetigo. At 12 months after mass drug administration, 1261 (77·6%) of 1625 people in the second set of ten villages had their skin examined, of whom 29 (2·3%) had scabies (relative reduction 88%, 95% CI 76·5–99·3) and 81 (6·4%) had impetigo (relative reduction 74%, 63·4–84·7). In the 3 months after mass drug administration, 10 614 attended outpatient clinics for any reason compared with 16 602 in the 3 months before administration (decrease of 36·1%, 95% CI 34·7–37·6), and during this period attendance for skin sores, boils, and abscesses decreased by 50·9% (95% CI 48·6–53·1).

**Interpretation:**

Ivermectin-based mass drug administration can be scaled to a population of over 25 000 with high efficacy and this level of efficacy can be achieved when mass drug administration for scabies is integrated with mass drug administration of azithromycin for trachoma. These findings will contribute to development of population-level control strategies. Further research is needed to assess durability and scalability of mass drug administration in larger, non-island populations, and to assess its effect on the severe bacterial complications of scabies.

**Funding:**

International Trachoma Initiative, Murdoch Children's Research Institute, Scobie and Claire Mackinnon Trust, and the Wellcome Trust.

## Introduction

Scabies is a parasitic skin disease that affects an estimated 200 million people worldwide.^1^ It is endemic in many tropical developing countries, especially in rural and remote communities where access to treatment is lacking and domestic overcrowding is common.^2^

Scabies causes skin inflammation with itch that is frequently severe, and often associated with bacterial skin infection caused by *Staphylococcus aureus* and *Streptococcus pyogenes* (impetigo).^3^ This infection can in turn lead to severe complications including septicaemia and post-streptococcal glomerulonephritis, and increasing evidence supports a causal link to acute rheumatic fever and rheumatic heart disease.^4^ In April, 2017, growing awareness of the burden of disease due to scabies led WHO to recognise it as a neglected tropical disease.^5^ A key next step is the development and implementation of effective population-level control strategies.

**Research in context**

**Evidence before this study**

Evidence supports scabies control using mass drug administration in small island communities. We searched PubMed with no language restrictions for publications between Jan 1, 1968, and April 30, 2018, using the terms “mass drug administration” and “scabies”. We identified two non-controlled studies that found mass drug administration using topical permethrin (San Blas Islands, Panama) or oral ivermectin (Lau Lagoon, Solomon Islands) was highly effective in the control of scabies. The skin health intervention Fiji trial (SHIFT), the first comparative trial of mass drug administration for scabies, observed a decrease in scabies prevalence of 94% in the ivermectin-based mass drug administration and 67% in the permethrin mass drug administration groups at 12 months after the intervention. However, all three of these trials included fewer than 1000 participants in the study groups. A large-scale trial of mass drug administration for endemic scabies has been advocated as a global health need, but never implemented.

**Added value of this study**

This study showed that ivermectin-based mass drug administration can be scaled to a population of over 25 000 people and achieve a similar efficacy as in small island populations. Moreover, this study showed that mass drug administration for the public health control of scabies can be integrated with azithromycin mass drug administration for trachoma, and additional health benefits were achieved, specifically decreases in outpatient attendance for multiple communicable disease syndromes.

**Implications of all the available evidence**

This study supports large-scale implementation of ivermectin-based mass drug administration for control of scabies in locations where the condition is identified as a public health priority. Further research questions include the durability in decreasing the prevalence of scabies and impetigo and the effect of mass drug administration on the complications of scabies and impetigo, cost-effectiveness, dosing regimens, and acceptability of the intervention to individuals and communities.

Standard guidelines for scabies control focus on treatment of individuals with symptoms, and their household contacts.^6^ Although this approach can achieve a high cure rate for affected individuals, reinfestation from untreated family and community members occurs rapidly in endemic settings, leaving the overall community prevalence unchanged.^7^

By contrast, the strategy of mass drug administration, involving treatment of entire communities, has been shown in small community-based trials^8^, ^9^ to substantially decrease the prevalence of both scabies and secondary impetigo. Several small, single-arm studies^8^, ^10^, ^11^ suggested the effectiveness of mass drug administration using topical permethrin or oral ivermectin,^8^ with ivermectin widely used in lymphatic filariasis and onchocerciasis elimination programmes.^11^ A report from Zanzibar, Tanzania,^12^ showed a decrease of over 68% in prescriptions of treatments for scabies after five annual rounds of mass drug administration with ivermectin and albendazole (given for lymphatic filariasis) in a population of over 1 million people. The first comparative trial of mass drug administration for scabies was the skin health intervention Fiji trial (SHIFT),^9^ which found that ivermectin-based mass drug administration decreased the prevalence of scabies by 94% 1 year after the intervention, and was superior to both mass drug administration of topical permethrin and standard care. However, the generalisability of this finding was restricted by the study's location in small, isolated populations (the ivermectin mass drug administration group had a baseline population of 716 people) and the authors recognised a need for a larger scale evaluation.^9^

High prevalences of scabies have been reported in the Pacific region, including in a population-based survey in Western Province, Solomon Islands, where the all-ages prevalence of scabies was 19·2% and impetigo was 32·7% in 2014.^13^ Other neglected tropical diseases, including trachoma, are also public health problems in the Solomon Islands.^14^ A national programme to eliminate trachoma was planned in all ten provinces that comprise the Solomon Islands and commenced in 2014, using the WHO SAFE (ie, surgery for trichiasis, antibiotics, facial cleanliness, and environmental improvement) strategy, which includes mass drug administration with azithromycin.^14^

We planned to use the azithromycin mass drug administration infrastructure to study the codelivery of ivermectin-based mass drug administration. In the azithromycin ivermectin mass drug administration (AIM) trial, we had the primary outcomes of safety and feasibility of large-scale mass co-administration of ivermectin and azithromycin and we recently reported these outcomes,^15^ in particular the absence of serious adverse events, in a study population of over 25 000 people. The secondary outcome focused on the efficacy of the intervention against community prevalence of scabies and impetigo, which we report in this Article.

## Methods

### Study setting and population

This prospective, single-arm, before-and-after, community intervention trial was undertaken in Choiseul Province of the Solomon Islands. The Solomon Islands is an island nation in the south Pacific with an estimated population of approximately 580 000 people in 2016,^16^ living largely in rural and remote settings,^17^ and has a Human Development Index rank of 156 of 188. The province of Choiseul occupies the entirety of one large and several smaller islands, with a population of 26 372 recorded in the 2009 national census.^16^ Health care is provided by 17 nurse aid posts, ten rural health clinics, one area health centre, and one hospital located in the provincial capital, Taro. Based on a 2013 population-based survey that showed a prevalence of active trachoma in children aged 1–9 years above the elimination threshold,^14^ Choiseul had been scheduled to receive azithromycin mass drug administration in 2015 through the national eye-health programme. These plans were modified to incorporate coadministration of ivermectin-based mass drug administration for scabies and impetigo.

We selected ten sentinel villages for a baseline skin survey. A full list of settlements on the island with their populations estimated by use of the most recent census was provided by the Ministry of Health and Medical Services. We used simple random sampling to select the sentinel settlements, with the only inclusion criterion of a population of 100–250 people. On the same basis, 1 year after the intervention we randomly selected ten different villages for the skin survey. At both timepoints, all residents of selected villages were invited to participate in the skin survey. Additionally, at 12 months, we randomly selected four of the initially selected sentinel villages and revisted them to evaluate efficacy in paired sites.

All residents of selected sentinel villages were eligible to participate in skin surveys for this study, with no age restrictions, and written informed consent was obtained from adults and from the parents or guardians of children.

The trial was registered with the Australian and New Zealand Trials Registry (ACTRN12615001199505) and approved by the Solomon Islands National Research Ethics Committee (15/33) and the Royal Children's Hospital Human Research Ethics Committee (35148A). An independent data safety monitoring committee oversaw the trial. Verbal consent was obtained from all individuals to participate in the mass drug administration programme.

### Procedures

Teams were assembled from local health staff and trained for both azithromycin-based and ivermectin-based mass drug administration. The AIM study team had extensive preliminary discussions with key stakeholders and followed an established process for the introduction of new community health initiatives in the Solomon Islands. This process included endorsement by the Solomon Islands Ministry of Health and Medical Services, and engagement with the communities involved. Information sheets explaining the study were distributed by trained community nurses and were available to study participants before enrolment.

The study coordinator (LR), who is highly experienced in scabies diagnosis, examined the skin of each participant for scabies and impetigo at both baseline and 12 months. One of two local nurses assisted with electronic data entry of examination findings using Open Data Kit (ODK, Seattle, USA). We used the same clinical diagnostic methods for these diseases as used in previous studies in Fiji and the Solomon Islands.^9^, ^18^ Scabies was defined as pruritic inflammatory papules with a typical anatomical distribution, such as the webs of the fingers, hands, wrists, and ankles, as per integrated management of childhood illnesses guidelines.^18^ Examination excluded breasts and genitals, unless requested by participants and then only in a separate, private examination area. Impetigo was defined as papular, pustular, or ulcerative lesions surrounded by erythema. The definition of crusted scabies we used was as previously described.^19^

All outpatient attendances at government clinics in the Solomon Islands are recorded in a standard case register (including patient age, sex, reason for attendance) at the clinic level. Each month, information collected in these registers is required to be transferred electronically via the District Health Information System (DHIS2) to the Solomon Islands Ministry of Health and Medical Services statistics office at the national level. Monthly reporting completeness is the proportion of clinics that submitted a report for that month. Because this proportion varied from month to month, we standardised absolute counts for Choiseul Province by assuming that those clinics that submitted a report were representative of all clinics in the province. We obtained data for the 12 months before and 12 months after the mass drug administration.

The regimen for trachoma antibiotic mass drug administration followed standard WHO guidelines,^20^ identical to the regimen used for mass drug administration for trachoma in the rest of the country. Participants were offered a single dose of 20 mg/kg azithromycin, up to a maximum of 1 g, using weight-based bands. Children weighing less than 12·5 kg received azithromycin oral suspension at a dose of 20 mg/kg. Drug administration was directly observed for all participants receiving azithromycin. Infants younger than 6 months received topical 1% tetracycline ointment for administration by a parent or guardian to both eyes twice per day for 6 weeks. A trained study nurse provided instructions to the parents on how to apply tetracycline eye ointment.

For ivermectin-based mass drug administration, participants were offered either two doses of oral ivermectin 200 μg/kg 7–14 days apart using weight-based bands, or if ivermectin was contraindicated (such as for pregnant and breastfeeding women and children weighing less than 12·5 kg) they were instead offered two applications of topical 5% permethrin cream 7–14 days apart. Drug administration was directly observed for all participants receiving ivermectin. Participants who were offered permethrin were given the option to have a trained nurse apply the cream in a private room at the clinic or to apply it themselves at home. The first dose of ivermectin or permethrin was administered at the same time as the antibiotic treatment.

### Outcomes

The main outcome of the study (a secondary outcome in the original AIM trial) was the prevalence of scabies and impetigo in the ten randomly selected villages at 12 months compared with ten different randomly selected villages at baseline. We chose a new set of villages at 12 months to ensure that our estimates of prevalence were as generalisable as possible to the whole province.

Additional analyses were comparison of the total number of people who attended outpatient clinics for any cause in the 3 months after mass drug administration, not including the month of administration, with the 3 months before mass drug administration. We chose a 3 month period because of the likelihood (as perceived by study investigators) that a clinically meaningful effect of mass drug administration at clinic level would persist for at least this period of time.^21^ We excluded analysis of data from the month during which mass drug administration was being delivered because the intervention was rolled out over 4 weeks and might have led to heightened clinic activity due to referrals by field teams for incidentally found medical issues. In addition to the 3-month comparisons, we compared attendance at outpatient clinics for any reason the 1 month before and after mass drug administration, the 12 months before and after mass drug administration (not including the administration month), and the 3-month period 12 months before mass drug administration with the 3-month period after mass drug administration.

Furthermore, we also returned to four of the baseline villages and compared the prevalence of scabies and impetigo at 12 months and baseline in these villages using paired analysis.

### Statistical analysis

On the basis of 2014 prevalence data from the Solomon Islands and SHIFT data,^9^, ^13^ we expected that the overall prevalence of scabies would be approximately 20% at baseline, decreasing to less than 5% after mass drug administration. We estimated that an overall sample size of 1200 individuals would be required at each of the two timepoints, assuming 80% participation and accounting for a 3% village-level correlation coefficient (assuming approximately 50–300 individuals per site at each timepoint). This sample size would allow us to estimate the prevalence at each timepoint with high precision (ie, within 5% or −5% absolute width of the respective CIs), and allow us to detect the difference in prevalence as significant at the 0·05 level when comparing the two timepoints (ie, 20% *vs* 5%) with 80% power. The statistical test of significance for this comparison, to be applied at the two-sided 0·05 level, was a χ^2^ test that takes into account the sample sizes at both the baseline and 12-month sampling points and accounts for cluster size.^22^ We calculated absolute (difference between baseline and month 12) and relative reductions (ratio of month 12 to baseline) in prevalence and their 95% CIs on the basis of their respective variances using the binomial distribution.^23^ We analysed the distribution of scabies and impetigo by demographic factors and calculated adjusted odds ratios (ORs) by fitting a logistic regression model adjusting for sex, age group, and village variables. We analysed clinic data by age group and by 12 communicable disease categories for which data are routinely reported. Clinic presentations were adjusted to monthly reporting completeness across all clinics in Choiseul Province by dividing reported cases by the monthly reporting rate (appendix).

We did all analyses using Stata version 14.2. This trial was registered with the Australian and New Zealand Trials Registry (ACTRN12615001199505).

### Role of the funding source

The funders of the study had no role in study design, data collection, data analysis, data interpretation, or writing of the report. The corresponding author had full access to all the data in the study and had final responsibility for the decision to submit for publication.

## Results

**Figure 1:**
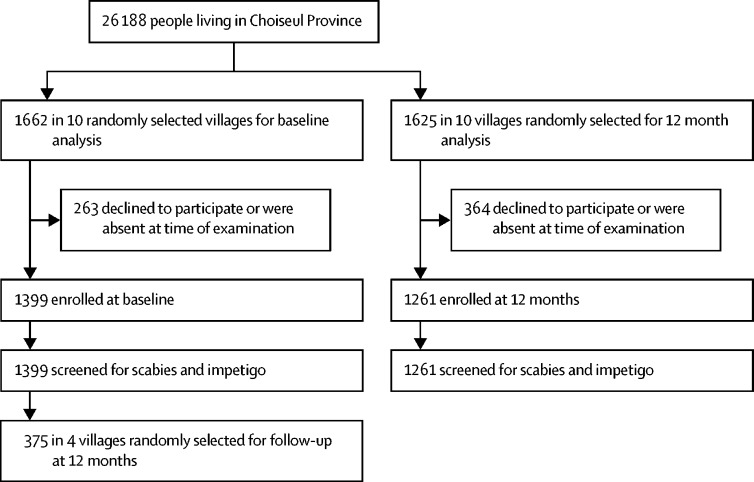
Study profile

**Figure 2:**
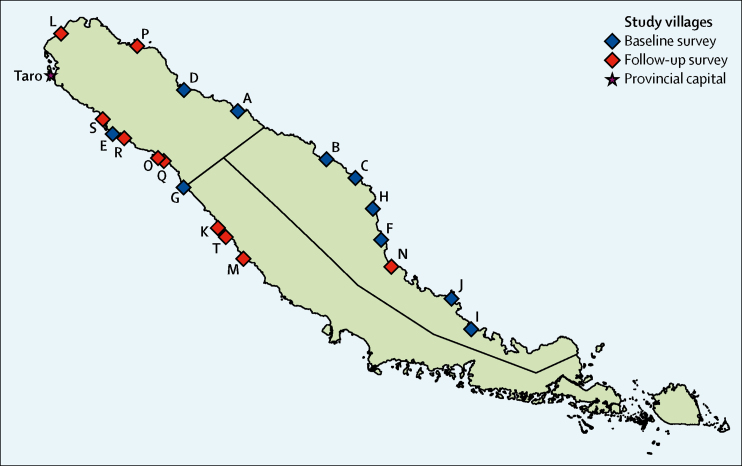
Villages selected for baseline and follow-up surveys in Choiseul Province, Solomon Islands

In September, 2015, over 4 weeks, 26 188 participants were enrolled, 99·3% of the resident population in the Choiseul Province based on the 2009 census (n=26 372).^16^ At baseline, of 1662 people living in the ten sentinel villages (villages A–J), 1399 (84·2%) consented to participate and were examined. 12 months after mass-drug administration, in September, 2016, of 1625 people in the second set of sentinel villages (villages K–T), 1261 (77·6%) consented to participate and were examined (figure 1). The sex and age distributions of individuals included in the two samples were similar (Table 1, Table 2) The locations of each village chosen, each designated with a letter from A to T, are shown on figure 2. At baseline, 209 (14·9%) of 1399 people in the ten baseline sentinel villages were ineligible to receive ivermectin, of whom 75 (36%) were children weighing less than the required 12·5 kg and 134 (64%) were pregnant or breastfeeding women, and so were offered permethrin.

**Table 1:** Characteristics of participants in ten baseline sentinel villages

		**Baseline population (n=1399)**
**Overall**		
Sex		
	Male	686 (49·0%)
	Female	713 (51·0%)
Age, years		
	Median	14 (7–34)
	<5	231 (16·5%)
	5–9	250 (17·9%)
	10–14	225 (16·1%)
	15–24	175 (12·5%)
	25–34	177 (12·6%)
	≥35	341 (24·4%)
**Village level enrolment**		
Village A		n=209
	Participants	178 (12·7%)
Village B		n=147
	Participants	114 (8·1%)
Village C		n=118
	Participants	70 (5·0%)
Village D		n=174
	Participants	157 (11·2%)
Village E		n=206
	Participants	171 (12·2%)
Village F		n=109
	Participants	106 (7·6%)
Village G		n=219
	Participants	162 (11·6%)
Village H		n=102
	Participants	88 (6·3%)
Village I*		n=118
	Participants	54 (3·9%)
Village J*		n=260
	Participants	299 (21·4%)

Data are median (IQR) or n (%) as a proportion of the study population.

* Residents of villages I and J were seen together because they were all attending a social gathering at the time of examination; combined enrolment for villages I and J: 353 (93·4%) of 378.

**Table 2:** Characteristics of participants in ten 12-month sentinel villages

		**Month 12 population (n=1261)**
**Overall**		
Sex		
	Male	570 (45·2%)
	Female	691 (54·8%)
Age, years		
	Median	15 (7–34)
	<5	155 (12·3%)
	5–9	229 (18·2%)
	10–14	209 (16·6%)
	15–24	227 (18·0%)
	25–34	130 (10·3%)
	≥35	311 (24·7%)
**Village level enrolment**		
Village K		n=129
	Participants	108 (8·6%)
Village L		n=186
	Participants	95 (7·5%)
Village M		n=199
	Participants	122 (9·7%)
Village N		n=121
	Participants	93 (7·4%)
Village O		n=165
	Participants	156 (12·4%)
Village P		n=118
	Participants	108 (8·6%)
Village Q		n=132
	Participants	111 (8·8%)
Village R		n=251
	Participants	229 (18·1%)
Village S		n=109
	Participants	70 (5·5%)
Village T		n=215
	Participants	169 (13·4%)

Data are median (IQR) or n (%) as a proportion of the study population.

In the ten baseline sentinel villages (villages A–J), 261 people had scabies (18·7%, 95% CI 16·7–20·8; table 3). Prevalence was highest in children aged 5–9 years (34·0%, 95% CI 28·1–40·2; adjusted OR 4·7, 95% CI 3·0–7·2, when compared with participants aged 35 years and older; appendix). Scabies was more common in girls and women than boys and men (adjusted OR 1·3, 95% CI 1·0–1·7; appendix). Scabies was observed in all ten baseline sentinel villages, with prevalences ranging from 15·1% to 31·5% (appendix). 347 people had impetigo (24·8%, 95% CI 22·6–27·1; table 3), and prevalence was highest among children aged 5–9 years (prevalence 46·4%, 95% CI 40·1–52·8; adjusted OR 7·8, 95% CI 5·1–12·0; appendix).

**Table 3:** Prevalence of scabies and impetigo at baseline and 12 months

	**Baseline**	**12 months**	**Absolute reduction in prevalence**	**Relative reduction in prevalence**
**Scabies**				
n/N	261/1399	29/1261	**..**	**..**
Prevalence	18·7% (16·7–20·8)	2·3% (1·6–3·3)	16·4% (14·2–18·6)	88% (76·5–99·3)
**Impetigo**				
n/N	347/1399	81/1261	**..**	**..**
Prevalence	24·8% (22·6–27·1)	6·4% (5·2–8·0)	18·4% (15·7–21·0)	74% (63·4–84·7)

Data are n/N and prevalence, with 95% CIs in parentheses.

At 12 months, in the second set of ten sentinel villages (villages K–T), 29 people had scabies (2·3%, 95% CI 1·6–3·3), corresponding to a relative reduction in prevalence of 88% (95% CI 76·5–99·3) from baseline (table 3). 81 people had impetigo (prevalence of 6·4%, 5·2–8·0; relative reduction of 74%, 95% CI 63·4–84·7; table 3). Full prevalence data for scabies and impetigo by age group and village at 12 months are in the appendix.

Among the four randomly selected villages examined at both baseline and 12 months, (villages A, B, C, and D) the decrease in prevalence of scabies and impetigo followed a similar pattern to that observed when comparing the two distinct sets of sentinel villages. Overall, scabies prevalence decreased from 19·5% (101 of 519) to 1·3% (five of 375; relative reduction of 93·3%, 95% CI 74·5–100) and impetigo from 22·4% (116 of 519) to 5·1% (19 of 375; relative reduction of 77·2%, 95% CI 58·0–96·0; appendix). We observed no cases of crusted scabies among participants.

In October to December, 2015, the 3 months after mass drug administration, 10 614 people attended outpatient clinics in Choiseul Province for any reason compared with 16 602 in June to August, 2015, the 3 months before mass drug administration (table 4), corresponding to a decrease of 36·1% (95% CI 34·7–37·6). We observed a similar decrease when we compared attendance in the 1 month periods before (August, 2015, n=6269) and after (October, 2015, n=3624) mass drug administration (decrease of 42·2%, 95% CI 41·0–43·4). All-cause outpatient attendance decreased from 19 789 in October to December, 2014, to 10 614 in October to December, 2015, showing a decrease of 46·4% (95% CI 45·7–47·1). 68 471 people attended outpatient clinics during the 12 months before mass drug administration (September, 2014, to August, 2015) and 59 162 attended in the 12 months after administration (October, 2015, to September, 2016, a decrease of 13·6% (95% CI 13·3–13·9).

**Table 4:** Outpatient all-cause clinic attendance in the 3 months before and after the intervention

		**June–August, 2015**	**October–December, 2015**	**Reduction in presentations**
Age group, years				
	0–<1	765	638	128 (16·7%)
	1–4	2618	1973	645 (24·6%)
	5–14	2733	1516	1217 (44·5%)
	15–49	7470	4429	3041 (40·7%)
	≥50	3016	2058	958 (31·8%)
Total		16 602	10 614	5989 (36·1%)

Data are n and n (% reduction).

The overall decrease in outpatient attendance for reported communicable diseases from the 3 months before to the 3 months after mass drug administration was 40·7% (95% CI 39·6–41·8, table 5). A larger decrease was observed for presentations categorised as skin sores, boils, and abscesses (50·9%, 95% CI 48·6–53·1) than for any other presentation of disease. When comparing the 3-month period 12 months before mass drug administration (October to December, 2014) with the 3-month period after (October to December, 2015), we observed a large decrease in attendance for communicable diseases (55·8%, 95% CI 54·8–56·7; appendix).

**Table 5:** Outpatient clinic attendance for communicable diseases syndromes in the 3 months before and after mass drug administration

		**June–August, 2015**	**October–December, 2015**	**Reduction in presentations (%)**
Presentation				
	Acute respiratory infection	4185	2673	1512 (36%)
	Skin sores, boils, and abscesses	1931	949	982 (51%)
	Acute ear infection	578	428	150 (26%)
	Watery diarrhoea	352	219	133 (38%)
	Red eye	239	161	78 (33%)
	Chronic ear infection	130	59	71 (55%)
	Yaws	114	15	99 (87%)
	Fungal skin infection	107	54	53 (50%)
	Bloody diarrhoea	58	14	44 (76%)
	Scabies	26	8	18 (69%)
	Neonatal bacterial infection	4	0	4 (100%)
Total		7723	4578	3144 (41%)

Data are n and n (% reduction).

## Discussion

We found the relative reduction in the prevalence of scabies was 88% compared with 94% in SHIFT,^9^ and the decrease in the prevalence of impetigo was substantial in both AIM and SHIFT (74% AIM *vs* 67% SHIFT). Hence, the results of the AIM trial reported here show that ivermectin-based mass drug administration can be scaled to a population of over 25 000 with a similar efficacy as in small island-based trial settings and that this level of efficacy can be achieved when mass drug administration for scabies is integrated with azithromycin mass drug administration for trachoma.

Given the absence of any known biological effect of ivermectin on bacterial organisms that cause skin infections, the substantial decrease in the prevalence of impetigo observed in AIM and SHIFT strongly support the hypothesis that scabies is an important causal factor for impetigo. The decrease in impetigo burden here exceeded estimates of the population-attributable risk of impetigo due to scabies in previous studies in the Pacific Islands.^13^, ^24^ The slightly greater decrease in prevalence of impetigo in AIM than in SHIFT, combined with the known activity of azithromycin against both *Staphylococcus aureus* and *Streptococcus pyogenes*,^25^ raises the possibility that azithromycin mass drug administration might have had an additional effect on the prevalence of impetigo beyond that of ivermectin-based mass drug administration alone. However, a community-based trial^26^ in the Solomon Islands in 2016–17 comparing ivermectin mass drug administration with ivermectin plus azithromycin mass drug administration found no difference in the decrease in prevalence of impetigo between the two groups at 12 months after the intervention (relative reduction 75% *vs* 73%; p=0·49).^26^

The AIM trial has shown that integration of mass drug administration for control of scabies and trachoma is feasible. However, other than a previous study in Zanzibar,^12^ few assessments of the effect of mass drug administration containing ivermectin for lymphatic filariasis and onchocerciasis on other neglected tropical diseases exist.^27^ Formal assessments of the effect of these programmes on scabies and other neglected tropical diseases, such as strongyloidiasis, would be of value in fully characterising the benefits of ivermectin mass drug administration, and the roll-out of triple therapy for control of lymphatic filariasis (ivermectin, diethylcarbamazine, and albendazole) provides a crucial opportunity to assess the effect of this regimen on scabies.^28^ The importance of making permethrin available for children and others who are contraindicated for ivermectin, as done in this study, needs to be assessed in these other mass drug administration programmes.

We observed a substantial effect of ivermectin and azithromycin mass drug administration on attendance at outpatient clinics, including attendance for skin sores, boils, and skin abscesses, yaws, acute respiratory infections, and diarrhoeal disease. These broader health benefits have been documented previously after mass drug administration of azithromycin for trachoma,^29^, ^30^, ^31^ including as documented in randomised trials that have found a decrease in overall mortality in young children.^29^, ^32^ Although the effect on acute respiratory infections and diarrhoeal disease observed in AIM is probably associated with azithromycin alone, the effect on attendance at outpatient clinics for bacterial skin infection might have been driven by both ivermectin and azithromycin.

To inform development of public health policies for effective scabies control, several outstanding research questions associated with implementation of mass drug administration for scabies exist, in addition to those associated with scalability and integration. These questions include the effect of mass drug administration on the complications of scabies and impetigo, including severe skin and soft tissue infections, sepsis due to *S aureus* and *S pyogenes*, glomerulonephritis, and rheumatic fever. Monitoring of resistance in bacteria after azithromycin mass drug administration and in parasites after ivermectin mass drug administration will be necessary to mitigate any unintended consequence of large-scale control programmes.^33^ Although small targeted studies might be necessary to answer questions about optimal ivermectin dosing, implementation research alongside large-scale roll-out of mass drug administration could be of use to assess the effect of mass drug administration on complications of scabies and impetigo, cost-effectiveness, community acceptability, staff training, and distribution logistics.

Our study had several limitations. Our study was non-randomised, employing a pragmatic before-and-after design appropriate for assessment of the effect of the intervention when delivered in such a large-scale (province-wide) setting, so we cannot say with certainty that factors other than the intervention did not influence our findings. Nevertheless, we are unaware of any other changes over the study period that could have accounted for a decrease in the prevalence of scabies and impetigo of the observed magnitude. With demographic characteristics of the population samples at the two timepoints being very similar, the difference in non-participation at 12 months (22·4%) versus baseline (15·8%) is unlikely to have influenced the findings. We chose ten distinct villages at baseline and 12 months for assessment to eliminate bias that might have been introduced through the presence of the study team. The additional paired analysis of the four villages seen at both baseline and 12 months supports our overall efficacy findings.

In the AIM trial, we offered two doses of ivermectin as part of mass drug administration, whereas in SHIFT^9^ a second dose was only provided to those who had clinical scabies because the SHIFT study team was able to screen all individuals within the small study population (n=716). Although some experts suggest^3^ that the use of environmental measures might play a role in decreasing reinfestation, we found that mass drug administration alone has high efficacy in decreasing community prevalence of scabies and impetigo.^3^ Because a second dose of ivermectin adds complexity and cost to a programme of mass drug administration, studies are ongoing to determine whether the same benefit can be achieved with a single dose of ivermectin for scabies control in highly endemic settings (ACTRN12618001086257 and ACTRN12617000738325). Finally, the data available to us for outpatient presentations were generated through routine reporting, which might have led to under-reporting of specific diseases, but the conditions for reporting were the same in the periods before and after the intervention. Front-line clinicians and health-informatics staff were not made aware in advance that the number of people attending clinics was being collected as part of this research.

In the AIM trial, ivermectin plus antibiotic mass drug administration was highly effective at decreasing scabies and impetigo prevalence at 12 months after the intervention in a population of over 25 000 people in a province in the Solomon Islands. Other substantial short-term health benefits were observed, with notable decreases in all-cause outpatient clinic attendance and presentations for multiple communicable diseases. Scabies was designated by WHO as a neglected tropical disease in April, 2017, and countries are now considering how they will seek to control the disease within a public health framework. The efficacy data presented here support large-scale implementation of ivermectin-based mass drug administration for control of scabies in areas where scabies is identified as a public health priority. Operational research should be undertaken alongside roll-out of such interventions, to assess durability in the decrease of scabies and impetigo and the effect of mass drug administration on the complications of scabies and impetigo, cost-effectiveness, and acceptability.
