# Supplementary material for: Efficacy of mass drug administration with ivermectin for control of scabies and impetigo, with coadministration of azithromycin: a single-arm community intervention trial
Source: Lancet Infect Dis. 2019 May;19(5):510–8. doi: 10.1016/S1473-3099(18)30790-4 (PMC6483975; doi:10.1016/S1473-3099(18)30790-4)
Supplement: Supplementary appendix [file mmc1.pdf]

THE LANCET
Infectious Diseases

Supplementary appendix

This appendix formed part of the original submission and has been peer reviewed. We post it as supplied by the authors.

Supplement to: Romani L, Marks M, Sokana O, et al. Efficacy of mass drug administration with ivermectin for control of scabies and impetigo, with coadministration of azithromycin: a single-arm community intervention trial. *Lancet Infect Dis* 2019; published online April 4. http://dx.doi.org/10.1016/S1473-3099(18)30790-4.

SUPPLEMENTARY APPENDIX

Efficacy of mass drug coadministration of ivermectin and azithromycin for control of scabies and impetigo: a single-arm community intervention trial

Contents

	Page
**Supplementary Table 1.** Monthly clinic reporting completeness across Choiseul Province between September 2014 and December 2016	2
**Supplementary Table 2.** Prevalence of scabies in the 10 sentinel villages at baseline by gender, age and village	3
**Supplementary Table 3.** Prevalence of impetigo in the 10 sentinel villages at baseline by gender, age and village	4
**Supplementary Table 4.** Prevalence of scabies in the 10 sentinel villages at 12 months by gender, age and village	5
**Supplementary Table 5.** Prevalence of impetigo in the 10 sentinel villages at 12 months by gender, age and village	6
**Supplementary Table 6.** Prevalence of scabies and impetigo in participants in four paired villages visited at both baseline and 12 months	7
**Supplementary Table 7.** Outpatient clinic attendance for reported communicable diseases in the three months after the intervention compared to the same period 12 months prior	8

Supplementary Table 1. Monthly clinic reporting completeness across Choiseul Province between September 2014 and December 2016 (*September 2015 was the month for roll-out of mass drug administration)

Month	Reporting Completeness
September 2014	88·5%
October 2014	65·4%
November 2014	65·4%
December 2014	72%
January 2015	82·6%
February 2015	85·7%
March 2015	100%
April 2015	95·5%
May 2015	91·3%
June 2015	95·7%
July 2015	95·7%
August 2015	78·3%
September 2015*	91·7%
October 2015	91·7%
November 2015	95%
December 2015	94·1%
January 2016	100%
February 2016	100%
March 2016	95·7%
April 2016	100%
May 2016	100%
June 2016	100%
July 2016	88·9%
August 2016	87·5%
September 2016	91·3%
October 2016	91·3%
November 2016	87%
December 2016	73·9%

Supplementary Table 2. Prevalence of scabies in the 10 sentinel villages at baseline by gender, age and village (CI: confidence interval; OR: odds ratio adjusted for gender, age and village)

**Factor**		**Sample**	**Participants with scabies**			**Adjusted OR (95% CI)**
		**N**	**n**	**%**	**95% CI**	
**Total**		1,399	261	18·7	16·7 – 20·8	
**Gender**	Female	713	118	16·6	13·9 – 19·5	1 (ref)
	Male	686	143	20·9	17·9 – 24·1	1 .3 (1·0 – 1·7)
**Age (years)**	<5	231	60	26·0	20·4 – 32·1	3·2 (2·0 – 5·0)
	5–9	250	85	34·0	28·1 – 40·2	4·7 (3·0 – 7·2)
	10–14	225	56	24·9	19·4 – 31·1	3·0 (1·9 – 4·8)
	15–24	175	11	6·3	3·2 – 11·0	0·6 (0·3 – 1·2)
	25–34	177	15	8·5	4·8– 13·6	0·8 (0·4 – 1·6)
	≥35	341	34	10·0	7·0 – 13·7	1 (ref)
**Village**	A	178	28	15·7	10·7 – 21·9	1 (ref)
	B	113	25	22·1	14·9 – 30·9	1·5 (0·8 – 2·8)
	C	70	12	17·1	9·2 – 28·0	1·1 (0·5 – 2·3)
	D	158	36	22·8	16·5 – 30·1	1·6 (0·9 – 2·7)
	E	171	30	17·5	12·2 – 24·1	1·1 (0·6 – 2·0)
	F	106	16	15·1	8·9 – 23·4	1·0 (0·5 – 1·9)
	G	162	28	17·3	11·8 – 24·0	1·1 (0·6 – 2·0)
	H	88	15	17·1	9·9 – 26·6	1·1 (0·5 – 2·2)
	I	54	17	31·5	19·5 – 45·6	2·5 (1·2 – 5·0)
	J	299	54	18·1	13·9 – 22·9	1·2 (0·7 – 2·0)

Supplementary Table 3. Prevalence of impetigo in the 10 sentinel villages at baseline by gender, age and village (CI: confidence interval; OR: odds ratio adjusted for gender, age and village)

		**Sample**	**Participants with impetigo**			**Adjusted OR (95% CI)**
		**n**	**n**	**%**	**95% CI**	
**Total**		1,399	347	24·8	22·6 -27·1	
**Gender**	Female	713	147	20·6	17·7 – 23·8	1 (ref)
	Male	686	200	29·2	25·8 – 32·7	1·6 (1·2 – 2·0)
**Age (years)**	<5	231	65	28·1	22·4 – 34·4	3·5 (2·2 – 5·6)
	5–9	250	116	46·4	40·1 – 52·8	7·8 (5·1 – 12·0)
	10–14	225	97	43·1	36·5 – 49·9	6·8 (4·4 – 10·6)
	15–24	175	20	11·4	7·1 – 17·1	1·2 (0·6 – 2·1)
	25–34	177	15	8·5	4·8 – 13·6	0·8 (0·4 – 1·6)
	≥35	341	34	10·0	7·0 – 13·7	1 (ref)
**Village**	A	178	42	23·6	17·6 – 30·5	1·1 (0·6 – 2·2)
	B	113	25	22·1	14·9 – 30·9	1·0 (0·5 – 2·1)
	C	70	15	21·4	12·5 – 32·9	1 (ref)
	D	158	34	21·5	15·4 – 28·8	1·0 (0·5 – 2·0)
	E	171	45	26·3	19·9 – 33·6	1·3 (0·7 – 2·5)
	F	106	22	20·8	13·5 – 29·7	1·0 (0·5 – 2·0)
	G	162	49	30·2	23·8 – 37·9	1·6 (0·8 – 3·1)
	H	88	20	22·7	14·5 – 32·9	1·1 (0·5 – 2·3)
	I	54	15	27·8	16·5 – 41·6	1·4 (0·6 – 3·2)
	J	299	80	26·8	21·8 – 32·2	1·3 (0·7 – 2·5)

Supplementary Table 4. Prevalence of scabies in the 10 sentinel villages at 12 months by gender, age and village (CI: confidence interval; OR: odds ratio adjusted for gender, age and village)

Factor		Sample	Participants with scabies			Adjusted OR (95% CI)
		N	n	%	95% CI	
**Total**		1,261	29	2·3	1·5 – 3·3	
**Gender**	Female	691	61	8·8	6·8 – 11·2	1
	Male	570	13	2·3	1·2 – 3·9	0·1 (0·5 – 2·1)
**Age (years)**	<5	155	15	9·7	5·5 – 15·5	5·4 (2·1 – 14·3)
	5–9	229	2	0·9	0·1 – 3·1	0·4 (0·1 – 2·2)
	10–14	209	0	0		
	15–24	227	5	2·2	0·7 – 5·1	1·1 (0·3 – 3·8)
	25–34	130	1	0·8	0·0 – 4·2	0·4 (0·0 – 3·3)
	≥35	311	6	1·9	0·7 – 4·2	1 (ref)
**Village**	K	108	1	0·9	0·0 – 5·1	0·8 (0·7 – 8·7)
	L	95	0	0		
	M	122	0	0		
	N	93	6	6·5	2·4 – 13·5	5·8 (1·1 – 29·1)
	O	156	6	3·8	1·4 – 8·2	3·3 (0·7 – 16·8)
	P	108	1	0·9	0 – 5·1	0·8 (0·1 – 8·7)
	Q	111	6	5·4	2·0 – 11·4	4·8 (0·9 – 24·1)
	R	229	2	0·9	0·1 – 3·1	0·7 (0·1 – 5·3)
	S	70	5	7·1	2·4 – 15·9	6·4 (1·2 – 33·9)
	T	169	2	1·2	0·1 – 4·2	1 (ref)

Supplementary Table 5. Prevalence of impetigo in the 10 sentinel villages at 12 months by gender, age and village (CI: confidence interval; OR: odds ratio adjusted for gender, age and village)

		Sample	Participants with impetigo			Adjusted OR (95% CI)
		n	n	%	95% CI	
**Total**		1,261	81	6·4	5·1 – 7·9	
**Gender**	Female	691	31	4·5	3·1 – 6·3	1 (ref)
	Male	570	50	8·8	6·6 – 11·4	2·1 (1·3 – 3·3)
**Age (years)**	<5	155	23	14·8	9·6 – 21·4	17·9 (5·3 – 60·6)
	5–9	229	28	12·2	8·3 – 17·2	14·4 (4·3- 47·9)
	10–14	209	15	7·2	4·1 – 11·6	7·9 (2·3 – 27·8)
	15–24	227	10	4·4	2·1 – 8·0	4·7 (1·3 – 17·4)
	25–34	130	2	1·5	0·2 – 5·4	1·6 (0·3 – 9·7)
	≥35	311	3	1·0	0·2 – 2·8	1 (ref)
**Village**	K	108	6	5·6	2·1 – 11·7	1·6 (0·5 – 5·1)
	L	95	6	6·3	2·4 – 13·2	1·8 (0·6 – 5·8)
	M	122	7	5·7	2·3 – 11·5	1·7 (0·5 – 5·0)
	N	93	11	11·8	6·1 – 20·2	3·6 (1·3 – 10·2)
	O	156	1	0·6	0 – 3·5	0·2 (0 – 1·5)
	P	108	9	8·3	3·9 – 15·2	2·5 (0·9 – 7·1)
	Q	111	15	13·5	7·8 – 21·3	4·2 (1·6 – 11·3)
	R	229	12	5·2	2·7 – 9·0	1·5 (0·6 – 4·1)
	S	70	8	11·4	5·1 – 21·3	3·5 (1·2 – 10·5)
	T	169	6	3·6	1·3 – 7·6	1 (ref)

Supplementary Table 6. Prevalence of scabies and impetigo in participants in four paired villages visited at both baseline and 12 months (CI: confidence interval)

Village		Prevalence at baseline (95% CI) (n/N)	Prevalence at 12 months (95% CI) (n/N)	Absolute reduction in prevalence (95% CI)	Relative reduction in prevalence (95% CI)
**A**	**Scabies**	**15·7%** (10·7 – 21·9) (28/178)	**1·3%** (0·2 – 4·6) (2/154)	**14·4%** (8·8 - 20·6)	**92%** (55·6 - 100)
	**Impetigo**	**23·6%** (17·6 – 30·5) (42/178)	**6·5%** (3·2 – 11·6) (10/154)	**17·1%** (9·5 – 24·5)	**73%** (40·4 - 100)
**B**	**Scabies**	**22·1%** (14·9 – 30·9) (25/113)	**2·3%** (0·6 -8·0) (1/44)	**19·8%** (8·2 – 28·6)	**90%** (37·1 - 100)
	**Impetigo**	**22·1%** (14·9 – 30·9) (25/113)	**0** (0/44)	**22·1%** (11·7 – 30·6)	**100%**
**C**	**Scabies**	**17·1%** (9·2 – 28·0) (12/70)	**2·4%** (0·3 – 8·4) (2/83)	**14·7%** (5·5 -25·4)	**86%** (32·1 – 100)
	**Impetigo**	**21·4%** (12·5 – 32·9) (15/70)	**2·4%** (0·3 – 8·4) (2/83)	**19·0%** (9·1 – 30·1)	**89%** (42·3 - 100)
**D**	**Scabies**	**22·8%** (16·5 – 30·1) (36/158)	**0** (0/94)	**22·8%** (15·7 - 30·0)	**100%**
	**Impetigo**	**21·5%** (15·4 – 28·8) (34/158)	**7·5%** (3·0 – 14·7) (7/94)	**14·0%** (5·0 - 22·1)	**65·1%** (23·0 - 100)
**Total**	**Scabies**	**19·5%** (101/519)	**1·3%** (5/375)	**18·2%** (14·5 - 21·8)	**93·3%** (74·5 – 100)
	**Impetigo**	**22·4%** (116/519)	**5·1%** (19/375)	**17·3%** (13·0 – 21·5)	**77·2%** (58·0 – 96·0)

Supplementary Table 7. Outpatient clinic attendance for reported communicable diseases in the three months after the intervention compared to the same period 12 months prior

	Attendance October to December 2014	Attendance October to December 2015	Reduction in attendance (%)
Acute respiratory infection	5754	2673	3081 (53·5)
Skin sores, boils and abscesses	2364	949	1415 (59·9)
Acute ear infection	710	428	282 (39·7)
Watery diarrhoea	634	219	416 (65·5)
Red eye	247	161	86 (34·8)
Yaws	175	15	160 (91·5)
Chronic ear infection	175	59	116 (66·4)
Fungal skin infection	156	54	102 (65·6)
Bloody diarrhoea	120	14	106 (88·4)
Scabies	18	8	10 (56·9)
Neonatal bacterial infection	0	0	0
**Total**	10352	4578	5774 (55·8)
